# The Attractions of Medicine

**Published:** 1895-09-07

**Authors:** 


### The Hospital

An Institutional Journal of

**The Medical Sciences and Hospital Administration,**

with

**AN EXTRA NURSING SUPPLEMENT.**

Vol. XVIII.—No. 467.] [Sept. 7, 1895.

### The Attractions of Medicine.

Every man who, having completed his studies and passed his examinations, has become a fully-admitted practitioner of physic, feels, among other symptoms of a rising *esprit de corps,* a strong desire to act as doorkeeper at the entrance gates of the profession. It is not that, having got inside himself, he would like to keep everybody else out. It is rather that he experiences a strong impulse to keep out certain classes of persons, and an impulse equally strong to draw in certain other classes.

Perhaps it must inevitably be the case that the majority of those who enter medicine, as of those who enter other learned professions, do so in order that they may earn a living and occupy the status of educated persons. These, however "respectable" they may be, add nothing to medicine except mere dull weight. They have to be endured, and carried, and governed, and guided by the rather limited number who have higher motives and better brains. Motives and brains constitute character. The man who has himself brains and character enough to really love his profession considers it a very significant part of his duty to get it occupied as far as he can with men of high motives and first-rate intellectual capacity.

For men of this class medicine has sterling and enduring attractions. To begin with, it is really a "learned profession" at the present time. What is more, its learning is chiefly the "new learning," the learning in which all the cultured world, and indeed all the world that thinks, is profoundly interested. No department of mental activity is so full of life and eager energy as that occupied by the new learning. Men want to know what Science has still to show them; what new thoughts she will encourage them to think; what new lines of individual, social, and political life she will have them enter upon. Whatever advantages there may be, and there are many, in being "up to date," quite abreast of what is newest and freshest in thought, speculation, and life, are shared in by medical science to the full.

Work and results are what medicine has to offer to the best young intellects of our period. It is only the most capable minds that ever master the broad groundworks of anatomy and physiology, developed as they are on the lines of organic chemistry and pathology, which constitute the true foundations of medicine. The majority acquire a smattering of all; they pass their examinations with a smattering; they practise throughout life with a smattering, which constantly grows less. Smatterers never know what medicine really is. The man of capacity who has mastered the broad facts and principles of the structure and functions of the human body has by his achievement relatively mastered the structure and functions of the whole of the vertebrate kingdom. He has thus begun to be a man of science in the true and philosophic sense of that term.

What does the intellectual young doctor build on this foundation? An "art of healing" first of all, if he intend to live by his calling. But much more also than a mere art of healing. He builds a knowledge of man, as man; of man at the apex of civilisation, and also of man at the base, when he was but little removed from the Simian type which still so closely resembles him in structure and form: and he bridges over the chasm between the two with the perennially interesting facts of historic development.

But the doctor who has really come at the inner soul of modern physic cannot be content with mere knowledge and speculation, however interesting these may be in themselves, any more than he can be content with the art of healing alone. His instinct —an instinct which no conventionalism or desire for material prosperity can check—is to put his scientific weapons to the proof. He desires to effect something—to heal those who are sick by all means, but also to modify and improve the natural and relatively healthy man in the mass. His aim is by philosophic teaching on such subjects as marriage, child-rearing, mental and physical education, work, recreation, morality, and religion, to aid in the evolution of a higher type of men and women—of a nobler, more conquering, more stable race.

These are great aims. They are not the cramped and narrow aims of the lawyer and the policeman, whose limited business it is to keep order in the civilised world. Nor are they even the aims of the religious teacher, who essays to make men truthful, honest, and single-minded. They are aims which include both these, but which go deeper than both. The modern doctor begins where Aristotle and the old Greek philosophers left off. It cannot be otherwise. Every man of real mental power who comes into physic, and there are many, feels that he must burst through the ancient prison walls of convention which would confine his powers within the limits of a mere "healing art." He gradually becomes conscious that nothing less than this is his proper business—to direct the development of the human race.

In our sober judgment these and their like are irresistible attractions to young men of a certain class. It is young men of this class, strong in mental powers, clear in intelligence, broad in philosophy, high in motive and aim, to which we desire to open wide the doors of modern medicine. What other calling has anything greater or nobler to offer? Wealth may not be easy of attainment in physic. It is much less easy of attainment in all callings than it was thirty years ago. But the science-loving young man to whom we have appealed is not so "money-mad" as many of the men of our generation. Science—that is, true science—despises the mere money-lover quite as much as religion does. No young man who loves truth and progress need hold back from medicine for fear of being disappointed with her. If he follows her with the genuine ardour with which any honest and capable man always follows the profession of his choice, he will say of her after years of experience what was said of Cleopatra, that "age cannot wither her, nor custom stale her infinite variety."

